# Concentrations of Boar Taint Compounds Are Weakly Associated with Sexual Behavior of Young Boars

**DOI:** 10.3390/ani12121499

**Published:** 2022-06-09

**Authors:** Elena Boschi, Sara Faggion, Chiara Mondin, Paolo Carnier, Valentina Bonfatti

**Affiliations:** Department of Comparative Biomedicine and Food Science, University of Padova, Viale dell’Università 16, 35020 Legnaro, Italy; elena.boschi@phd.unipd.it (E.B.); chiara.mondin.3@phd.unipd.it (C.M.); paolo.carnier@unipd.it (P.C.); valentina.bonfatti@unipd.it (V.B.)

**Keywords:** pigs, libido, indole, skatole, androstenone

## Abstract

**Simple Summary:**

Boar taint is an off-odor and off-flavor affecting pork meat caused by the accumulation of three main compounds in the adipose tissue of uncastrated male pigs: androstenone, skatole, and indole. Boar taint occurrence is associated with sexual maturity onset; thus, strategies for reducing boar taint compound concentrations might lead to impaired male sexual behavior. This study investigated the relationships between boar taint compound concentrations and sexual behavior traits (sexual arousal, salivation, mounting performance, interest in the dummy sow, penis unsheathing, and overall libido) scored during training with the dummy sow in 391 young boars. Overall, our results suggest that a weak relationship between boar taint compounds and sexual behavior traits exists. The maximization of libido performances was observed at high levels of androstenone, intermediate or low levels of skatole, and intermediate to high levels of indole, but the relationship between BT compounds and sexual behavior appears to be weak.

**Abstract:**

Strategies to control boar taint (BT) in meat relies on the reduction of skatole, indole, and androstenone concentration. This might have unfavorable effects on the libido of breeding boars. The association between BT compound concentration in backfat and libido was investigated in 391 commercial breeding boars. Six sexual behavior traits (SBT; sexual arousal, salivation, mounting performance, interest in the dummy sow, penis unsheathing, and overall libido score) were scored during the training of the boars with the dummy sow. Variation in SBT was analyzed by proportional-odds cumulative logistic models. Overall, indole, skatole, and androstenone concentrations were weakly associated with libido. Farm of origin, age at training or body weight, and BT compound levels were poor predictors of boar performance (the area under the ROC curve ranged from 0.60 to 0.69). This indicates that BT compound concentrations were weakly associated with libido, even though the probability of observing good SBT scores increased with high levels of androstenone, intermediate or low levels of skatole, and intermediate to high levels of indole. Hence, practices aiming at reducing androstenone, and controlling the concentrations of skatole and indole to intermediate levels are not expected to impair the libido of young boars.

## 1. Introduction

Despite being beneficial for reproductive success, the increased concentration of testicular steroids implicated in semen production and libido could be associated with an increase in the synthesis of undesired compounds responsible for the development of boar taint (BT), an off-odor and off-flavor typical of pork from adult, uncastrated, male pigs [1,2]. Boar taint is mainly caused by the accumulation in fat tissue of androstenone, a sexual hormone synthesized in Leydig cells in testes and involved in testosterone metabolism, and skatole and indole, two fecal compounds resulting from tryptophan degradation carried out by gut bacteria, even though many other substances have been reported to contribute to a lesser degree [1]. 

Puberty plays a key role in regulating androstenone levels in entire male pigs by the stimulation of the neuroendocrine system which leads to increased biosynthesis of testicular steroids [3]. In sexually mature boars, androstenone level is strictly related to the ability of the individual to synthesize this steroid. The rise in androstenone level occurring at puberty is followed by a rise in skatole and indole levels in blood serum at first and later in the fat tissue. The amount of skatole and indole formed in the hindgut is primarily determined by the availability of tryptophan, which largely depends on diet composition [4]. The amount of skatole and indole in the serum and adipose tissue is also partially regulated by testicular steroids [2]: a major source of tryptophan derives from the cell debris resulting from the turnover of the intestinal epithelium [5,6] and a high turnover rate is associated with high levels of insulin-like growth factor 1 (IGF1), in turn controlled by 17β-estradiol. Increased levels of testicular steroids, namely androstenone and 17β-estradiol, suppress hepatic skatole and indole degradation by inhibiting the enzymes responsible for their metabolism [4,7].

A strategy to avoid the risk of BT is slaughtering entire male pigs before puberty. However, this is not a viable option in pigs intended for protected designation of origin dry-cured ham production, as minimum age at slaughter dictated by product specifications to guarantee compliance for processing is after sexual maturity [8,9]. Hence, surgical castration of male piglets is a common practice [2].

In recent years, to meet the increasing request of banning surgical castration in the EU, breeding companies have become progressively interested in selective strategies aimed at decreasing the risk of BT. The attempts at reducing BT in pork by selective breeding might potentially lead to an unfavorable outcome in other traits of interest for commercial farms, such as libido and semen quality in male pigs intended for reproduction purposes [10,11]. Previous attempts at selecting against androstenone resulted in decreased performance and sexual maturation due to decreased levels of androgens and estrogens. In gilts of a low-androstenone line, delayed puberty was observed [12].

Currently, there is very limited evidence on the relationship between BT compound concentrations and sexual behavior in entire male pigs. Hence, the aim of this study was to investigate the relationship between BT compound concentrations in fat tissue and sexual behavior traits in young commercial breeding boars.

## 2. Materials and Methods

### 2.1. Animals and Sample Collection

The study included all the commercial young boars of the purebred C21 Goland sire line (Gorzagri, Fonzaso, Italy) that were trained with the dummy sow from December 2019 to November 2021. The boars (*n* = 391) had an age ranging from 6 to 9 months and were from two different farms. The number of animals from each farm was 290 and 101, respectively. The concentration of androstenone, skatole and indole (hereafter referred to as “BT compounds”) was assessed approximately 1 month before the animals started the training with the dummy sow, during which sexual behavior was tested. 

### 2.2. Quantification of Boar Taint Compounds

Animals were subjected to local anesthesia and a subcutaneous fat sample (~0.5 g) was collected from the neck area using the biopsy device (SUISAG, Sempach, Switzerland) described in [13]. Samples were collected and stored at −80 °C until laboratory analyses.

The concentration of androstenone, indole and skatole was assessed by reversed-phase HPLC with fluorescence detection. Sample preparation prior to HPLC analysis was performed according to the method described by [14]. An Agilent 1260 HPLC Infinity system (Agilent Technologies, Santa Clara, CA, USA), controlled by the LC OpenLAB software (Agilent Technologies, Santa Clara, CA, USA), was used for analysis. The system consisted of a 1260 quaternary pump (G1311), an autosampler (G1329B, 1260 autosampler-ALS) equipped with a thermostat (G1329B, 1290 Infinity thermostat), a column thermostat (G1316A, 1260 TCC), and a fluorescence detector (G1321C, 1260 FLD). Chromatographic separations were performed using a C18 column (Zorbax Eclipse Plus, 95 Å, 5 µm, 4.6 × 100 mm, Agilent Technologies, Santa Clara, CA, USA) with a porous silica base, equipped with a security guard column cartridge (Phenomenex, Torrance, CA, USA).

Two different chromatographic separations, one for indole and skatole, and one for androstenone, were performed. In both separations, the column temperature and flow rate were maintained at 47 °C and 1.5 mL/min, respectively. For indole and skatole, an isocratic separation was performed with the mobile phase consisting of 20% of solvent A (0.1% of TFA in acetonitrile) and 80% of solvent B (30% acetonitrile in H_2_O). The total time of the chromatographic separation was 5 min. The excitation and emission wavelengths of the fluorescence detector were 270 and 350 nm, respectively. Injection volume was 10 µL.

Androstenone concentration was quantified after sample derivatization following the method described by [14]. Chromatographic conditions were the following: from 0 to 2 min, 5% solvent A, 60% solvent B, and 35% solvent C (MeOH); from 2 to 2.5 min, linear increase of solvent C to 75% and linear decrease of solvent B to 20%; from 2.5 to 8.5 min, linear increase of solvent C to 78.5%, linear decrease of solvent A to 18.3% and linear decrease of solvent B to 3.2%; from 8.5 to 8.6 min, linear increase of solvent C to 100%; then 100% solvent C for 1 min; finally, the initial chromatographic conditions were restored in 0.1 min and maintained until the end on the analysis (11 min). The excitation and emission wavelengths of the fluorescence detector were 346 and 521 nm, respectively. Injection volume for androstenone quantification was 20 µL.

The detection limit of the analysis was 0.08 µg/g for androstenone, 1.21 ng/g for skatole, and 1.34 ng/g for indole. The residual coefficient of variation (CV) of compound concentration across days (calculated on 5 samples injected over 5 consequent days as a measure of reproducibility) was <5.2%, whereas the residual CV of compound concentration within day of analysis (calculated on 5 samples injected 5 times on the same days as a measure of repeatability) was <2.6%.

### 2.3. Assessment of Sexual Behavior Traits

Sexual behavior was assessed during the training of the young boars with the dummy sow. During the training period, animals were housed in individual pens. The time interval between the transfer of the animal to the individual pen and the training session ranged from 1 to 30 days, depending upon the farm management and commercial needs. For each young boar, the training consisted of three mounting sessions with the dummy sow. Boars were permitted to attempt the mounting freely for 10 min. If the boar did not show any interest in the dummy sow or mounting intent within 10 min, the trainer stimulated the boar manually.

In the third training session, six sexual behavior traits (SBT) were appraised in the young boars using a linear grading system with scores ranging from 1 (poor) to 5 (good). The six SBT were: sexual arousal, defined as the excitement showed by the animal when the dummy sow was introduced in the pen; salivation, jaw chomping stimulating saliva production; mounting performance, defined as the promptness to mounting; interest in the dummy sow, defined as the promptness in approaching the dummy sow with nose, biting and sniffing; promptness of penis unsheathing; and overall libido score, a general score for the training session which was a combination of all the aforementioned traits. The R^2^ contribution of each SBT to the overall libido score, assessed using the function calc.relimp of the relaimpo package [15] in the R software (version 1.3.1073, Vienna, Austria) [16], was predominant for mounting promptness (40.7%) and penis unsheathing (30.8%), moderate for interest in the dummy sow (19.2%) and less important for sexual arousal and salivation (5.8 and 3.5%, respectively). 

Overall libido scores equal to 1 were attributed to animals failing the mounting. Due to the very low frequency (≤3.7%) of such score for all the SBT, scores 1 were grouped to scores 2; thus, SBT scores in the final statistical analysis ranged from 1 to 4.

The duration of the training session (DTS) was computed as the time interval from the introduction of the dummy sow in the pen to the end of the training session. While the assessment of sexual arousal, salivation, and interest in the dummy sow was performed shortly after the introduction of the dummy sow, and focused on the instinctive reaction of the boar to the dummy, scores for penis unsheathing, mounting performance, overall libido score, and DTS were influenced also by the intensity of the stimulation performed by the trainer and by ejaculation time. All animals were trained and scored by one experienced trainer.

### 2.4. Statistical Analysis

#### 2.4.1. Sexual Behavior Traits

The relationship between BT compound concentrations and SBT was investigated by proportional-odds cumulative logistic models. The models were fitted to the data using the clm function of the ordinal package [17] in the R software (version 1.3.1073, Vienna, Austria) [16]. A cumulative link model is a model suitable for the analysis of ordinal observations, i.e., observations represented as assignments into one of a finite set of C mutually exclusive categories following a natural order. In this study, for each SBT, C = 4. Ordinal observations can be represented by a random variable Y_i_ that takes a value c if the ith ordinal observation is an assignment into the c^th^ ordered category where c = 1, …, C and C ≥ 2. Such variable has a multinomial distribution $Y_{i}\sim \mathrm{Multinomial}\;\left(N,\;\pi_{1},\ldots ,\pi_{C}\right)$ where $\pi_{1},\;\ldots ,\;\pi_{C}$ are probabilities of assignment into category 1, …, C. Then, the cumulative probability for $Y_{i}$ of being less than or equal to a specific category c = 1, …, C − 1 is:

(1) $$P\left(Y_{i}\leq c\right)=\pi_{1}+\ldots +\pi_{c}$$

and the cumulative odds of assignment into category c or lower categories can be defined as:

(2) $$odds=\frac{P\left(Y_{i}\leq c\right)}{P(Y_{i}>c)}$$

for c = 1, …, C − 1 since $P\left(Y_{i}>C\right)=0$.

By assuming that assignment into a category depends upon the value of a latent continuous variable L and specific threshold cutoff points $\theta_{1},\;\ldots ,\theta_{c-1}$ on L such that:

$$\begin{array}{c} Y_{i}=1{\;\mathrm{if}\;L}_{i}\leq \theta_{1}{\;\mathrm{else}\;Y}_{i}>1,\\ Y_{i}\leq \;2{\;\mathrm{if}\;L}_{i}\leq \theta_{2}{\;\mathrm{else}\;Y}_{i}>2,\\ \ldots ,\\ Y_{i}\leq \;C-1{\;\mathrm{if}\;L}_{i}\leq \theta_{C-1}{\;\mathrm{else}\;Y}_{i}=C, \end{array}$$

and that the probability $P\left(L_{i}>\theta_{c}\right)$ is given by the logistic function, the proportional odds cumulative logistic model is:

(3) $$\log\left(\frac{P\left(Y_{i}\leq c\right)}{P(Y_{i}>c)}\right)=\theta_{c}-\overset{k}{\underset{1}{\sum }}\beta_{k}X_{\mathrm{ki}}$$

where, for each SBT, θ_c_ represents the threshold cutoff point, *k* is the number of covariates (fixed effects) included in the model, $\beta_{k}$ is a regression coefficient, and X_ki_ is a regression variable. In this study, the effects accounted for by the model were those of the farm, of indole, skatole and androstenone concentration class, of body weight (for sexual arousal only) and of age at training (for penis unsheathing and overall libido only). The data of each BT compound were categorized into 3 classes of equal size based on the percentile of the compound concentration distribution. Class 1 included boars with a low concentration, below the 33rd percentile of the distribution; class 2 included records between the 33rd and 67th percentile, corresponding to an intermediate level; class 3 included animals above the 67th percentile, corresponding to a high concentration of the compound in fat. The categorical effects (i.e., concentration class of each BT compound and farm) were accounted for in the model through the use of dummy variables (2 dummy variables for the intermediate and high concentration class of each BT compound, respectively, and 1 dummy variable for farm 2). Hence, the estimated regression coefficients for such variables, obtained by solving model (2), quantify the change in the logarithm of the cumulative odds relative to the reference class (i.e., the low concentration class of each BT compound and farm 1) and mean value for body weight and age at training (when applicable).

To assess the occurrence of problems due to multicollinearity among the explanatory variables, estimates of parameters for models including the effect of a single BT compound separately were compared in preliminary analyses with those obtained for models accounting for the effects of all the three BT compounds jointly. As results across models were consistent, the final models for each SBT included the effects of indole, skatole and androstenone concentration jointly. The effect due to occurrence and intensity of the stimulation operated by the trainer was not considered in the model as it was a criterion for the assessment of libido and influenced the score assigned by the trainer.

The odds-ratio (OR) for each covariate in model (3) was computed as follows:

(4) $$OR_{X_{k}}=\exp\left({\hat{\beta }}_{k}\right)$$

where ${\hat{\beta }}_{k}$ is the estimated regression coefficient for the covariate.

For each SBT, the probability of assignment into each one of the four scoring categories, as resulting from different features of the animal (farm, concentration class of each BT compound, age at training and body weight), was computed as:

$$\begin{array}{c} P\left(Y_{i}=1\right)=\frac{1}{1+e^{-({\hat{\theta }}_{1}-\sum_{1}^{k}{\hat{\beta }}_{k}X_{\mathrm{ki}})}},\\ P\left(Y_{i}=2\right)=\frac{1}{1+e^{-({\hat{\theta }}_{2}-\sum_{1}^{k}{\hat{\beta }}_{k}X_{\mathrm{ki}})}}-P\left(Y_{i}=1\right),\\ P\left(Y_{i}=3\right)=\frac{1}{1+e^{-({\hat{\theta }}_{3}-\sum_{1}^{k}{\hat{\beta }}_{k}X_{\mathrm{ki}})}}-P\left(Y_{i}=1\right)-P\left(Y_{i}=2\right),\\ P\left(Y_{i}=4\right)=1-P\left(Y_{i}=1\right)-P\left(Y_{i}=2\right)-P\left(Y_{i}=3\right), \end{array}$$

where ${\hat{\theta }}_{1},{\hat{\theta }}_{2},{\hat{\theta }}_{3}$ are estimated threshold cutoff points and ${\hat{\beta }}_{k}$ are estimated regression coefficients.

The cumulative link model assumes that the thresholds are constant for all values of the independent variables, which is generally referred to as the proportional odds assumption. A likelihood ratio test of the proportional odds assumption for indole, skatole and androstenone concentration class, farm, age at training and body weight, was performed as in [18], with no evidence that the proportional odds assumption was violated.

#### 2.4.2. Androstenone, Skatole, and Indole Levels as Predictors of Boar Performance

The multiclass area under the receiving operating characteristics curve (AUC) was used to evaluate, for each SBT, the performance of the proportional-odds cumulative logistic model in discriminating between scoring categories, in a 5-fold cross-validation. An AUC value is expected to be closer to 0.5 when the performance of a classifier is equal to a classification strategy based on random guessing, whereas when AUC is greater than 0.5, the classifier performs better than random guessing. Values of AUC close to 1 indicates a perfect classes separation [19]. Multiclass AUC as defined by [20] was computed in R using the package *pROC* [21].

#### 2.4.3. Duration of the Training Session

The distribution of DTS was skewed, with the majority of the animals concluding the session shortly after the introduction of the dummy sow in the pen. Hence, DTS was log-transformed before the statistical analysis to make the distribution of the trait approximately normal. The association between DTS and BT was then investigated by a linear model using the *lm* function of the *stats* package in R [16]. The linear model was the following:

(5) $$y_{\mathrm{ijklm}}=\mu +F_{i}+{\mathrm{AND}}_{j}+{\mathrm{SKA}}_{k}+{\mathrm{IND}}_{l}+\varepsilon_{\mathrm{ijklm}}$$

where y_ijklm_ is an observation of the log-transformed DTS, µ is the model intercept, F_i_ is the fixed effect of farm i (i = 1,2), AND_j_ is the fixed effect of class j of androstenone (j = 1, …, 3), SKA_k_ is the fixed effect of class k of skatole (k = 1, …, 3), IND_l_ is the fixed effect of class l of indole (l = 1, …, 3) and $\varepsilon_{\mathrm{ijklm}}$ is a random residual.

## 3. Results and Discussion

### 3.1. Androstenone, Skatole, and Indole Concentrations

The average live weight and age at fat biopsy collection were 157.2 ± 15.5 kg and of 213 ± 13 days, respectively. The sample means and range of the BT compound concentrations for each of the three classes (low, intermediate, high) are reported in Table 1. Average values of androstenone were in the range reported in the literature, whereas contents of skatole and indole were lower than those reported previously for heavy pigs [14,22] and consistent with results reported for pigs of 100–130 kg body weight [13,23]. Possible factors explaining this low indolic compound deposition include intestinal flora, dietary composition, and housing. Boar taint compound concentrations were not normally distributed, with a large majority of pigs exhibiting low concentrations of BT compounds, as observed in previous studies [14,24,25].

### 3.2. Correlations between Variables

Pearson product–moment correlations between body weight at fat sampling, age at training, and log-transformed BT compound concentrations are reported in Table 2. The correlations between the concentration of BT compounds and age at training or body weight were not statistically different from zero, with the only exception of the correlation between body weight and androstenone (*r* = 0.13, *p*-value = 0.01). These results are consistent with the literature [26]. The age at which the rise in steroid levels occurs depends largely on the variability in the sexual development process but, when all animals are sexually mature, the amount of circulating androstenone depends on the potential for androstenone production [25,27]. As evidenced by [27], in young light boars both age and body weight exert significant effects on variation in fat androstenone. In older and heavier boars, a higher proportion of animals are sexually mature and express their potential for androstenone production. Thus, age effects become less important while live weight still affects androstenone concentrations, even though the correlation between fat androstenone level and body weight is low [26].

Conversely, variation in skatole and indole levels has been reported to be significantly affected by age. However, different breeds exhibit distinct age-related distribution of plasma skatole and indole levels [24]. Hence, the correlation between age and concentration of indolic compounds might vary depending on the breed and age.

Correlations between BT compound concentrations were all positive and ranged from moderate to high, in agreement with those reported in previous studies [13,24,28]. The correlation was moderate between androstenone and the other BT compounds, and high (*r* = 0.70; *p*-value < 0.001) between contents of indole and skatole. This is to be ascribed to the similar nature of the latter compounds and their common pathway [4].

### 3.3. Distribution of Scores for Sexual Behavior Traits and Duration of the Training Session

**Figure 1:**
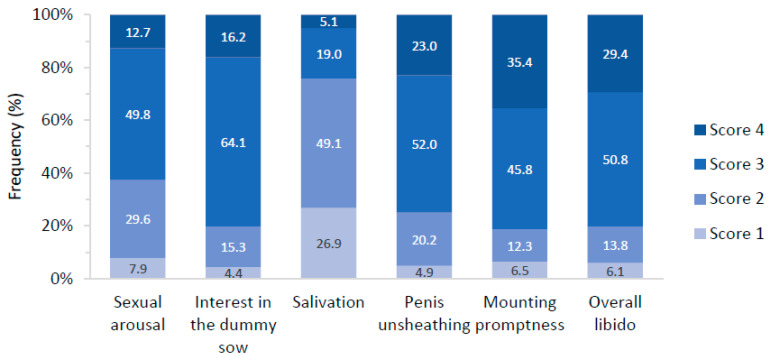
Distribution (%) of the scores (1 = poor; 4 = good) for the sexual behavior traits evaluated during the last session of training of the boars with the dummy sow.

Age at training with the dummy sow was, on average, 233 ± 21 days. Average scores of the SBT were generally high (on average 4.04 ± 0.86 for the overall libido score, data not reported in tables). Relative frequencies of the scores for each of the SBT are depicted in Figure 1. Mounting promptness was the trait exhibiting the highest proportion of animals with a score equal to 4 (35.4%), whereas salivation was the one having the least proportion of that score (5.1%). Score 3 was predominant for all the SBT (45.8–64.1%), except for salivation, whose predominant score was 2 (49.1%). The proportion of score 1 was low for all SBT (4.4–7.9%) except for salivation (26.9%). The proportion of boars that performed the mounting with no stimulation was very large and equal to 91% (results not reported in tables). These results can be attributed to the fact that libido was assessed at the end of training with the dummy sow.

Generally, lower SBT scores were associated with longer DTS (results not reported in tables). The training session lasted on average 14 min, but DTS varied largely (from 5 to 49 min). The frequency of pigs with DTS ≤ 15 min was equal to 74.2%, whereas the proportion of animals whose training session lasted longer than 25 min was 6.6%.

### 3.4. Effect of Farm, Body Weight, and Age on Sexual Behaviour Traits

**Figure 2:**
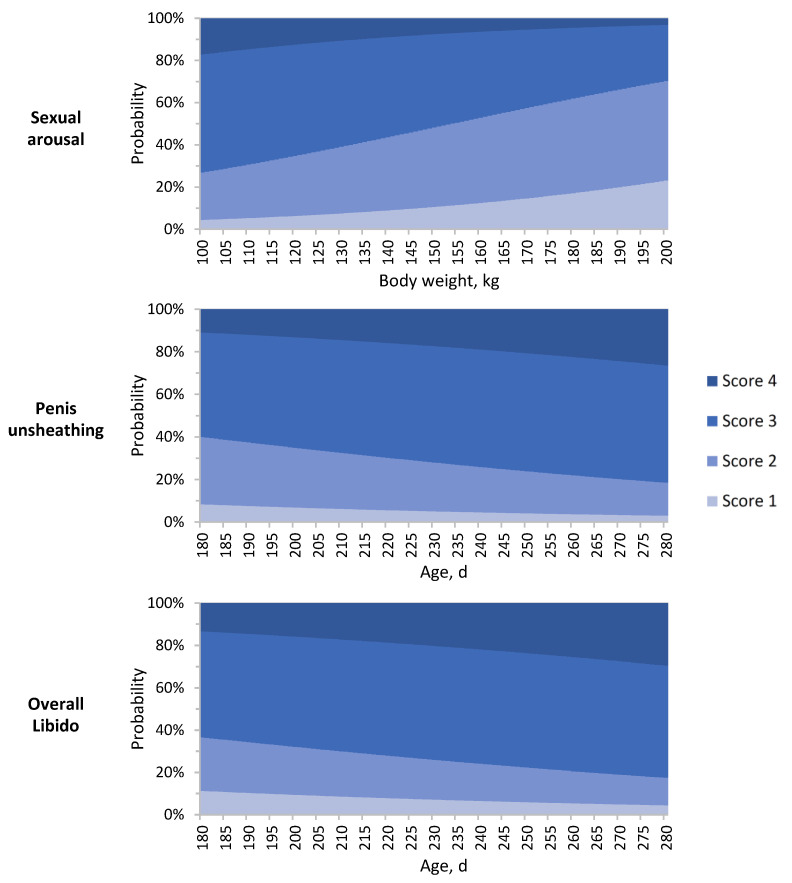
Probability of sexual arousal, penis unsheathing, and overall libido scores (1 = poor; 4 = good) at different body weight and age.

The farm had a significant effect on SBT, in agreement with findings of other studies [29,30,31]. The effect of the farm on the SBT might be attributed to the effect of different environmental factors and husbandry conditions including, for example, temperature and housing system. Body weight (estimated at constant androstenone, skatole, and indole concentration) did not affect any of the SBT, except sexual arousal (OR = 0.98, *p*-value < 0.01). The probability of assignment into each sexual arousal scoring class, as affected by variation in body weight, is reported in Figure 2. A linear decrease in the probability of class 3 and 4 was observed at increasing body weight: at 100 kg body weight, the probability of assignment into class 3 and 4 was 55.9% and 16.9%, respectively. Such probabilities decreased to 26.6% and 3.1% at 200 kg body weight. As boars were fed ad libitum, heavier animals most likely corresponded to individuals characterized by greater growth potential and higher feed intake, which is partially regulated by sexual hormones: high levels of testicular androgens and estrogens have a suppressive effect on feed intake [32], which may lead to boars showing lower body weight and better libido than those of greater body weight. Excessive weight gains can also decrease the activity of the animals by making them fatter and lethargic, leading to compromised locomotive soundness and balance and impaired physical ability of a boar to mount a sow [33].

Age effects (estimated at constant androstenone, skatole, and indole concentration) affected significantly penis unsheathing and overall libido scores. Scores for these traits had similar patterns and their probability of being in a higher ordinal category increased at increasing age (OR = 1.01 for both traits, *p*-value < 0.05), as shown in Figure 2. In younger boars, the probability of class 1 for penis unsheathing and overall libido were 8.3 and 11.2%, respectively, and decreased in older boars to 3.0 and 4.5%, respectively. It can be hypothesized that older animals, in this age range investigated, had a higher testicular steroid concentration in plasma [22,34], and, consequently, a greater libido than younger animals. 

### 3.5. Association between Boar Taint Compound Levels and Sexual Behavior Traits 

**Figure 3:**
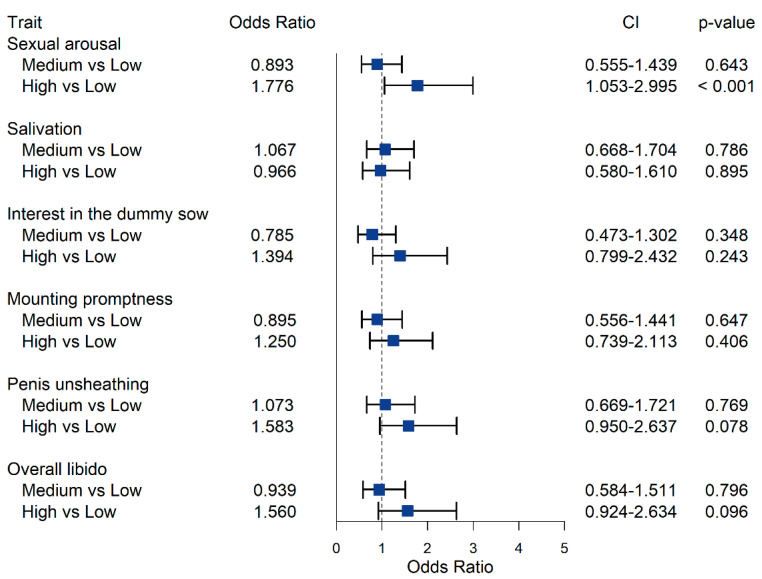
Odds ratio for the effect of average and high androstenone concentration in subcutaneous adipose tissue, compared to low androstenone concentration, on sexual behavior traits evaluated during the third training session. The dashed line corresponds to an odds ratio equal to 1.00.

**Figure 4:**
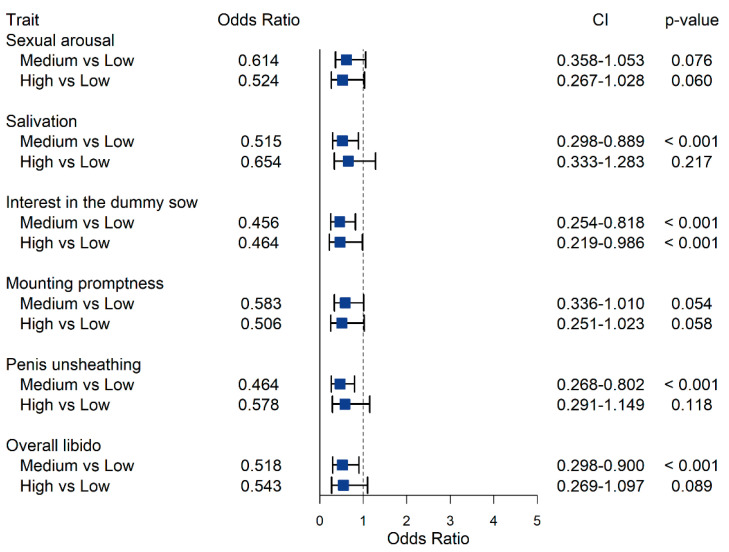
Odds ratio for the effect of average and high skatole concentration in subcutaneous adipose tissue, compared to low skatole concentration, on sexual behavior traits evaluated during the third training session. The dashed line corresponds to an odds ratio equal to 1.00.

**Figure 5:**
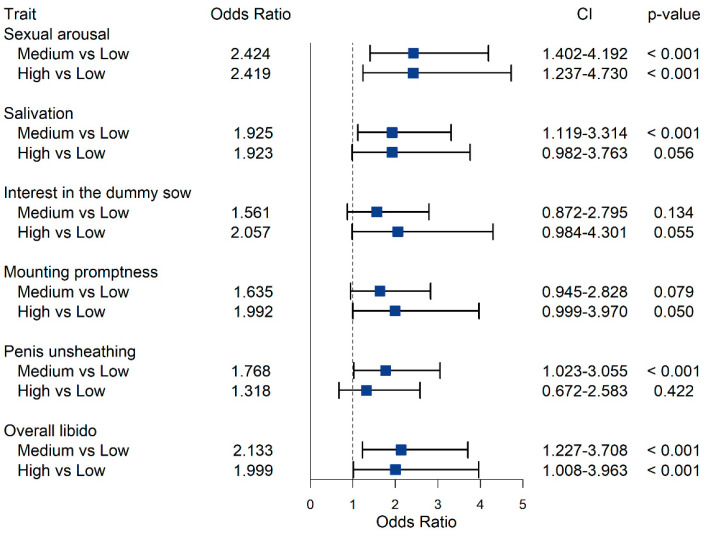
Odds ratio for the effect of average and high indole concentration in subcutaneous adipose tissue, compared to low indole concentration, on sexual behavior traits evaluated during the third training session. The dashed line corresponds to an odds ratio equal to 1.00.

**Figure 6:**
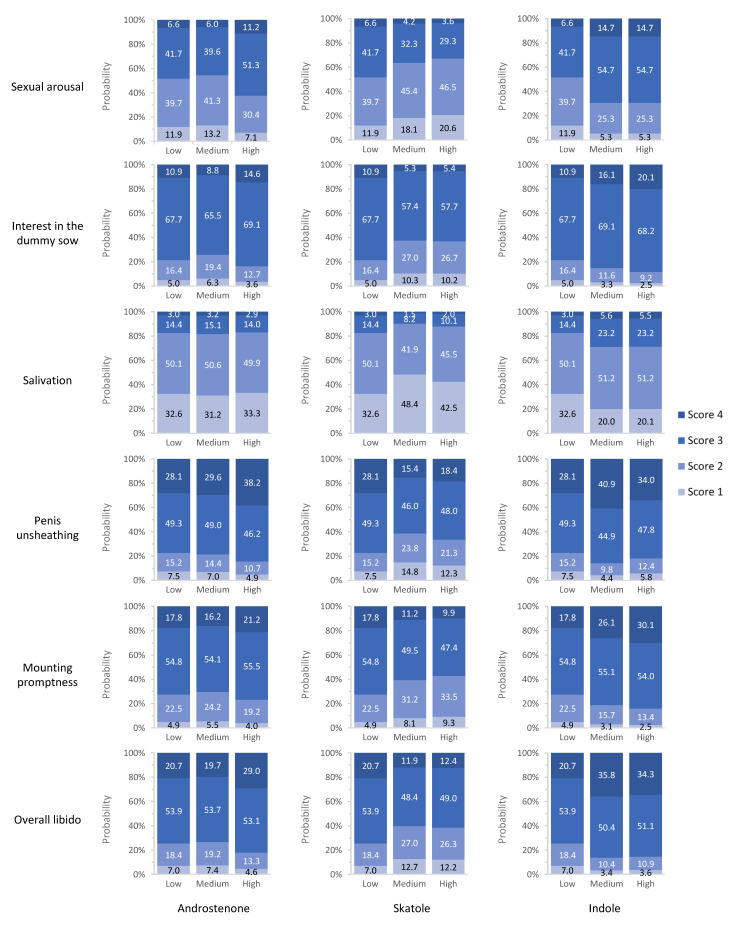
Probabilities of score 1 to 4 at low, intermediate, or high levels of each boar taint compound (at constant low level of the others) for the investigated sexual behavior traits.

Estimates of OR for intermediate and high androstenone, skatole and indole concentration class, compared to the class of low concentration, OR confidence intervals (CI) and *p*-values, are reported in Figure 3, Figure 4 and Figure 5, respectively. While the estimated OR for the class of intermediate concentration did not differ significantly from the one of low concentration (Figure 3), high androstenone levels in fat had OR > 1 for all SBT traits, except for salivation (OR = 0.97; *p*-value = 0.90). However, only the OR for sexual arousal (1.78; CI: 1.05–3.00) was statistically different from 1 (*p*-value < 0.05); this indicates that pigs with high androstenone concentration in subcutaneous adipose tissue were roughly 1.8 times more likely to be assigned into higher ordinal categories for sexual arousal compared to animals exhibiting low androstenone concentrations. At constant low levels of skatole and indole, the probability of class 4 for sexual arousal was 6.6% for boars with low androstenone, and it increased to 11.2% for boars with high androstenone (Figure 6). 

Our results indicate that the relationship between androstenone and SBT is generally weak. An association between BT and circulating sexual hormone concentrations has been reported [2]. However, the association between sexual hormone concentrations and BT was determined either by in-vivo studies, where concentrations of steroids were higher than physiological levels, or by comparing entire males with immunocastrated or surgically castrated males, a setting that largely increases the variability in serum steroid concentration, beyond its biological range. Conversely, our study focuses on entire males, where the variation in androstenone concentration is to be ascribed only to individual variation across animals. This might explain the lack of a significant association with boar performances observed in our study.

To the best of our knowledge, the relationship between androstenone (and skatole) concentrations and libido has been investigated only once, in a boar semen production center [22], where libido was assessed as the number of training sessions required to obtain a successful semen collection, and the number of mount refusals. Even though differences in the relationship between the investigated traits (steroid hormone concentrations, BT compound concentrations in fat, semen quality traits, and libido) were observed across genetic lines, no relationship was detected between androstenone or skatole concentration at 280 days and libido. However, the study focused on a limited number of animals (*n* = 93) and the reduced sample size might have affected the results.

In contrast with the results observed for androstenone, intermediate and high SKA concentration classes exhibited OR < 1, indicating that animals tended to exhibit poor libido compared to boars with low skatole (Figure 4). As OR estimates ranged from 0.46 to 0.65, animals with intermediate and high concentrations of skatole were approximately 2 times less likely to exhibit good scores for the SBT compared to animals exhibiting low levels of skatole. For salivation, penis unsheathing, and overall libido, scores assigned to boars with low levels of skatole were not significantly different from those of boars with high skatole levels, whereas intermediate skatole concentrations were significantly associated with lower scores than low skatole concentrations. For penis unsheathing, in boars with intermediate skatole compared to those with low skatole, the probability of assignment into class 4 decreased from 28.1% to 15.4%, while the probability of class 1 increased from 7.5% to 14.8% (Figure 6). Increased skatole levels were also significantly associated (*p*-value ≤ 0.05) with a decreased probability of interest in the dummy sow; in particular, the OR estimated for intermediate and high levels of skatole was 0.46. The probability of scores equal to 4 decreased from 11% to 5.3–5.4% in boars with intermediate or high skatole compared to boars with low skatole concentration in fat.

The estimated OR for indole were all greater than 1 and generally greater than those estimated for androstenone (Figure 5). Odds ratios for the class of high indole concentrations ranged from 1.32 for penis unsheathing to 2.42 for sexual arousal. This indicates that boars with high indole concentration were from 1.3 to 2.4 times more likely to exhibit greater libido scores than low-indole boars. Intermediate and high concentrations of indole were associated with increased odds for sexual arousal (*p*-value < 0.001) and overall libido (*p*-value < 0.001) in comparison with low indole concentrations. In particular, the probability of assignment into a class equal or greater than 3 for sexual arousal was 48.4% for boars with low indole content and 69.4% for boars with intermediate or high indole concentration (Figure 6). For penis unsheathing, high indole levels resulted in the same odds as for low indole levels. This indicates that high contents of indole did not result in a faster unsheathing than that observed in boars with intermediate indole levels. Conversely, intermediate indole levels were significantly associated with an increased probability of high penis unsheathing scores relative to low indole contents (OR = 1.77; *p*-value < 0.001). The probability of observing a score equal to 4 was 40.9% in boars with intermediate indole content and 28.1% or 34% in boars with low or high indole levels, respectively (Figure 6).

Overall, our results indicate that, while scores for sexual behavior increased at intermediate or high levels of indole, they also increased at intermediate or low levels of skatole. Hence, despite being synthesized and metabolized in the liver by the same enzymatic system [1], and being highly correlated, skatole and indole concentrations showed opposite associations with SBT. The controversial results obtained in the literature on the association between skatole and sexual steroids and the scarcity of studies on indole metabolism make the interpretation of this result difficult.

A competitive effect between skatole and indole for their synthesis or degradation, or a different control of steroids on one or more enzymes responsible for their synthesis or degradation might be hypothesized, but additional studies are necessary to elucidate these mechanisms.

While androstenone concentration and its accumulation in fat are related to testosterone synthesis and, consequently, to the concentration of serum steroids, contents of skatole and indole in fat are the result of several independent steps and largely rely on the balance between the rate of formation from tryptophan (mediated by 17β-estradiol) and absorption in the hindgut, and metabolic degradation in the liver. Increased 17β-estradiol levels, as well as increased androstenone levels, are involved in the suppression of hepatic degradation of skatole and indole [7]. Hence, high skatole and indole concentrations are expected when steroid concentrations are high. However, some studies indicate that testosterone is not involved in the regulation of skatole levels (e.g., [7]), suggesting that skatole might not be associated to sexual behavior.

While factors affecting indole are not fully known yet, skatole has been the focus of several studies as its concentration plays a major role on BT occurrence [1]. Nevertheless, results on the associations between skatole and sexual steroid concentrations, determined at puberty onset, are still inconsistent. 

Some authors hypothesized that the relationship between puberty and skatole (and consequently, also between libido and skatole) might be time-dependent, as the increase in skatole concentrations is delayed compared to the rise of testicular steroid levels [34]. This might indicate a possible breed-specific relationship between skatole and libido. Indeed, a few studies suggested a breed-dependent regulation of testicular steroids on hepatic skatole metabolism [35] and a breed- and time-dependent relationship between skatole levels and puberty. In particular, Babol et al. [24] observed that skatole levels in Yorkshire, Landrace, Hampshire, and Duroc pigs are relatively low up to 180–190 days of age, then increase later and are maintained high up to 240–360 days of age, depending on breed. Later, they decrease due to a more stable function of the intestine or the liver or both, which is a feature of mature pigs. Those authors observed also a considerable percentage of immature morphology in spermatozoa of Hampshire pigs, the breed in which skatole levels are maintained high for a longer time, suggesting that this pattern may justify the delayed puberty of this breed. In these circumstances, it is likely to observe a negative relationship between skatole concentration and the onset of puberty. 

In addition, if high steroid levels suppress the degradation of the indolic compounds [7], they might at the same time interfere with the formation of indolic compounds, by decreasing voluntary feed intake [36] and reducing the available amount of skatole and indole in the hindgut. Moreover, entire male pigs fed ad libitum might exhibit increased androstenone levels in fat compared to animals fed restrictively [37], likely because high energy availability might accelerate puberty [25], but high energy availability has also been reported to lower the formation of skatole in the hindgut [4]. All these factors suggest that the relationship between indolic compounds and steroid levels is complex.

Overall, results of our study indicate that the adoption of practices aiming at controlling the concentration of skatole and indole to intermediate levels is not expected to result in a decreased libido, and, in addition, can be favorable for consumer acceptability of pork meat, as some studies have shown that the consumer acceptability threshold for androstenone is in the range of 2–3 μg/g when skatole is present at a level lower than 0.1 µg/g [38,39]; that skatole threshold is about four times higher than the average skatole concentration estimated in this study (0.026 µg/g).

### 3.6. Androstenone, Skatole, and Indole Levels as Predictors of Boar Performance

Despite the significant OR of BT compounds on some SBT, in cross-validation, the AUC values of models including farm of origin, age at training or body weight, and BT compound levels, were moderate: 0.691 for sexual arousal, 0.601 for salivation, 0.677 for interest in the dummy sow, 0.679 for mounting promptness, 0.684 for penis unsheathing and 0.673 for overall libido. These values were slightly higher than 0.5, which corresponds to the expected value of AUC if SBT scores were attributed by random guessing. Hence, our results indicate that farm of origin, age at training or body weight, as well as BT compound levels, are poor predictors of boar performance. In addition, when the effect of BT compound levels was excluded from the models, AUC values decreased by a limited extent. In particular, the AUC values for the reduced models were the following: 0.65 for sexual arousal, 0.52 for salivation, 0.63 for interest in the dummy sow, 0.60 for mounting promptness, 0.64 for penis unsheathing and 0.63 for overall libido. Overall, this confirms that BT compounds are weakly associated with the sexual behavior of young boars. The AUC values of models including or not including the BT compound levels as predictors differed noticeably only for salivation and mounting promptness. It has to be noted that results are based on a relatively small number of animals, in which libido was evaluated by a categorical scoring system, and where the frequency distribution of the scores was unbalanced, all factors potentially affecting the predictive performance of models.

### 3.7. Association between Boar Taint Compound Levels and Duration of the Session

The effect of the concentration class of BT compounds on DTS was not statistically significant (Table 3). The R^2^ of the model was very low (3%), confirming that BT compound concentrations had a minor role on the variation in DTS. Almost 50% of the variability in DTS was due to the stimulation operated by the trainer, and the remaining variation could be ascribed to animal individual variation (data not reported in tables), suggesting a major role of individual experience or behavior on this trait. The lack of association between BT compound concentrations and DTS might be explained also by the fact that, in this study, measurement of DTS included also the ejaculation time, which is frequently used as an indicator of libido [40]. Reports on the duration of ejaculation in the boar vary from 3 to 20 min [41], so a long ejaculation time, which may be an indicator of good libido [42,43], can lead to increased overall DTS when compared with short ejaculation or no ejaculation.

## 4. Conclusions

Our results suggest that an association between androstenone, skatole, and indole concentrations in fat and sexual behavior in boars exists. The probability of observing good sexual behavior scores increased with high levels of androstenone, intermediate or low levels of skatole, and intermediate to high levels of indole. However, these relationships were generally weak, and practices aiming at reducing androstenone, or controlling the concentrations of skatole and indole to intermediate levels are not expected to exert negative repercussions on libido of young boars. The causal relationship between androstenone, skatole and indole and libido deserve further investigation. 

Our study evidenced that the specificity of the animals plays a major role in the variation observed. This might be partly due to the limited experience of the boars at the time of sexual behavior assessment or to the scoring system used.

Further studies are required to investigate the genetic correlations between androstenone, skatole and indole concentration and libido, with the aim to determine the feasibility of selective breeding programs, and identify the most appropriate strategy to reduce BT while ensuring reproductive success.

## Figures and Tables

**Table 1:** Mean (range) concentration of boar taint compounds in each class of concentration (low—below the 33rd percentile of the distribution; intermediate—between the 33rd and 67th percentile, high—above the 67th percentile).

Boar Taint Compound	Class of Concentration		
	Low	Intermediate	High
Androstenone (µg/g)	0.42 (0.08–0.62)	0.85 (0.64–1.17)	2.11 (1.17–8.28)
Skatole (ng/g)	8.65 (0.35–14.66)	25.57 (14.76–42.40)	116.83 (43.00–605.04)
Indole (ng/g)	4.76 (1.34–7.62)	12.69 (7.65–19.32)	40.20 (19.40–191.20)

**Table 2:** Pearson product-moment correlations between body weight at fat sampling, age at training with the dummy sow, and log-transformed boar taint compound concentrations ^1^.

Trait	Body Weight	Log (AND)	Log (SKA)	Log (IND)
Age	0.252 ***	0.068	0.097	0.055
Body weight		0.126 **	0.017	−0.043
log(AND)			0.309 ***	0.389 ***
log(SKA)				0.695 ***

^1^ log (AND)—log-transformed androstenone concentration; log (SKA)—log-transformed skatole concentration; log (IND)—log-transformed indole concentration; ** *p*-value ≤ 0.01; *** *p*-value ≤ 0.001.

**Table 3:** ANOVA table for the duration of the training session (*n* = 391).

Effect	DF	MS	F	*p*
Farm	1	1.39	10.27	0.001
Androstenone concentration class	2	0.20	1.45	0.236
Skatole concentration class	2	0.15	1.09	0.338
Indole concentration class	2	0.12	0.88	0.415

DF—degrees of freedom; MS—mean square.

## Data Availability

Restrictions apply to the availability of these data. Data were obtained from Gorzagri (Fonzaso, Italy) and are available from the authors with the permission of Gorzagri.
